# Comparing intra-abdominal pressures in different body positions via a urinary catheter and nasogastric tube: a pilot study

**DOI:** 10.1186/2110-5820-2-S1-S11

**Published:** 2012-07-05

**Authors:** Nirooshan Rooban, Adrian Regli, Wendy A Davis, Bart L De Keulenaer

**Affiliations:** 1 Intensive Care Unit, Fremantle Hospital, 1 Alma Street, 6160 Fremantle, Western Australia, Australia; 2 Medical School, The University of Notre Dame, Fremantle, Western Australia, Australia; 3 School of Medicine and Pharmacology, University of Western Australia, Fremantle Hospital, Western Australia, Australia

**Keywords:** intra-abdominal hypertension, intra-abdominal pressure, abdominal compartment syndrome, gastric pressure, body position

## Abstract

**Objectives:**

Intra-abdominal pressure (IAP) is most commonly measured via the bladder with the patient in the supine position. In the ICU, patients are nursed with the head of the bed elevated at 30° (HOB30) to reduce the risk of ventilator-associated pneumonia. This study investigated whether gastric pressure at HOB30 can be used as a surrogate measure of IAP via the bladder in the supine position.

**Methods:**

A prospective observational study was conducted in a single-centre intensive care unit. A total of 20 patients were included. IAP was recorded simultaneously via the bladder catheter (bladder pressure, IBP) and via nasogastric tube (gastric pressures, IGP) in the supine and HOB30 position. Each patient had three sets of IAP measurements performed at least 4 h apart.

**Results:**

In the supine position, mean IBP was 12.3 ± 4.5 mmHg compared to IGP of 11.8 ± 4.7 mmHg. The bias between the two groups was 0.5 and precision of 3.7 (LA, -6.8 to 7.5 mmHg). At 30 degrees, mean IBP was 15.8 ± 4.9 mmHg compared to IGP of 13.1 ± 6.1 mmHg. The bias between both groups was 2.7 with a precision of 5.5 (LA, -8.0 to 13.5). Comparing IBP in the supine position with IGP at 30° showed a bias of -0.8 and precision of 5.6 (LA, -10.1 to 11.6 mmHg).

**Conclusion:**

IAP measured via a nasogastric tube was less influenced by changing the body position from supine to HOB30 than was bladder pressure.

## Introduction

Intra-abdominal hypertension (IAH) and abdominal compartment syndrome (ACS) are associated with increased morbidity and mortality in critically ill patients [1,2]. As a result, evidence-based consensus definitions and recommendations for resuscitation and management of IAH and ACS have been developed [3,4]. The general approach is based on four principles: (a) serial IAP monitoring, (b) medical management to reduce IAP, (c) goal-directed optimisation of systemic perfusion and organ function and (d) early surgical decompression for IAH/ACS refractory to these interventions. Cheatham and Safcsak showed that implementing these recommendations including serial IAP monitoring and early abdominal decompression at an IAP of 25 mmHg significantly increased patient survival to hospital discharge from 50% to 72% [5].

The World Society of Abdominal Compartment Syndrome (WSACS; http://www.wsacs.org) recommends the measurement of IAP via the bladder in the supine position at end-expiration, ensuring that abdominal contractions are absent and with the transducer zeroed at the level of the mid-axillary line [3,4]. Intermittent and continuous measurement of IAP via the stomach, bladder and peritoneal cavity have been validated and used when this method is contra-indicated [6].

In the intensive care unit, most ventilated patients are nursed with the head of the bed elevated at 30° or 45° to reduce the risk of ventilator-associated pneumonia and gastric reflux [7,8]. Measuring IAP in the semi-recumbent position has been shown to adversely affect IAP readings when measured via the intravesical route [9,10]. The aim of our study was to compare the influence of body position, specifically head of bed elevation at 30° (HOB30), on bladder and gastric pressure. Furthermore, we examined the correlation between the intrabladder pressures (IBP) and intragastric pressures (IGP) in the supine and HOB30 position. Our hypothesis was that IGP at 30° are not influenced by the descent of abdominal contents in the semi-recumbent position and therefore would represent IBP in the supine position.

## Materials and methods

The Human Research Ethics Committee of Fremantle Hospital approved this prospective pilot study.

### Patient selection

Patients who were admitted to the Intensive Care Unit at Fremantle Hospital between December 2009 and April 2010 were consecutively enrolled in the trial. Patient inclusion criteria required them to be older than 18 years of age, to be sedated and mechanically ventilated and to have both a nasogastric tube and indwelling urine catheter in place. Reasons for exclusion were recent oesophageal, gastric or bladder surgery; patients who were moribund and unlikely to survive 24 h; patients who were pregnant and patients who were unable to lay flat for any reason. Patients were sedated to a Richmond Agitation and Sedation Scale of -5 (RASS) to ensure that abdominal muscle contractions were absent. Written informed consent was obtained by the next of kin as determined by the ethics committee.

### Bladder pressure

Once the patient was enrolled, IBP was measured according to the WSACS consensus recommendations using the standard bladder technique [3]. The IBP was measured through the patient's indwelling catheter, according to the modified Kron technique using an AbViser 300 or AbViser 611 kit (AbViser, Wolfe Tory Medical, Salt Lake City, UT, USA) [11,12]. The transducer was zeroed on the mid-axillary line at the level of the superior iliac crest. After 20 ml of normal saline was injected through the indwelling urine catheter, the IAP was measured at end-expiration in millimetres of mercury. The pressure transducer was connected to the electronic monitoring equipment available in the intensive care unit.

### Gastric pressure

Gastric pressures were measured via a standard nasogastric tube. Prior to measurement, the nasogastric tube was aspirated for air or gastric contents, and its position was confirmed by chest X-ray. A three-way stopcock was attached between the nasogastric tube and a standard pressure monitoring line. A Luerlok syringe was attached to one port of the stopcock for the instillation of 100 ml of sterile saline from the closed pressure monitoring system to ensure a continuous column of fluid between the stomach and the pressure transducer. A point level with the xiphisternum on the mid-axillary line was marked, and the transducer was zeroed at that level. This position corresponds to the position of the stomach allowing accurate measurement of gastric pressure in different body positions. After each measurement, the instilled volume of saline was then aspirated out of the nasogastric tube.

### Protocol

Each set of measurements included IBP and IGP in the supine position followed by the semi-recumbent position at HOB30. All four measurements were taken over a period of 5 to 10 min or less to reduce the possibility of changes in IAP over time. Three sets of readings were taken at least 4 h apart over a 24-h period.

### Data collection

Once patients were enrolled in the study, data on patient demographics including age, sex, weight, height, body mass index, diagnosis at admission, co-morbidities and presence of IAH risk factors were collected. Severity of illness for the 24-h period before the first IAP measurement was documented through calculation of the Acute Physiology and Chronic Health Evaluation score II, Simplified Acute Physiology Score II and Sequential Organ Failure Assessment Scores. For each set of IAP readings, mean arterial pressure, positive end-expiratory pressure and RASS were recorded. All study data were recorded on a case report form on paper and subsequently entered as de-identified patient data on a secure Microsoft Access database.

IAH was defined by a sustained or repeated pathological elevation in IAP ≥ 12 mmHg, whilst ACS was defined as a sustained IAP > 20 mmHg that is associated with new organ dysfunction/failure [3].

### Statistical analysis

Statistical analysis was performed using Medcalc (Medcalc version 9.3.5.0, Mariakerke, Belgium) and PASW Statistics 18 (SPSS Inc., Chicago, IL, USA). Data are presented as proportions (with 95% confidence interval as appropriate) or mean ± standard deviation (SD). Paired Student's *t *tests were used to test for statistical significance between two different measures of pressure on the same patients at the same times. General linear modelling (GLM) for repeated measures was used to test whether differences between the same measures of pressure on the same patients changed significantly over time. A significant level of *p *< 0.05 was used throughout. For assessing agreement between two methods of measurement of IAP, we used Bland-Altman plots [13]. The WSACS recommends a bias below 1 mmHg and a precision (defined as the standard deviation of the bias) of 2 mmHg, or thus, limits of agreement of -4 to +4 mmHg are necessary for two IAP techniques to be considered equivalent [14]. Pearson correlation plots were also utilised to assess agreement between the two methods. The Pearson correlation (*r*^2^) is +1 in the case of a perfect positive (increasing) linear relationship, and as it approaches zero, there is less correlation.

## Results

There were 20 patients enrolled in the study with a total of 240 IAP measurements. Patient demographics and severity of illness are presented in Table 1. One patient presented with an ACS, and of the remaining 19 patients, nine presented with IAH (47.4%). Sixty-five percent of the patients were medical, 30% were surgical and one patient (5%) was enrolled after trauma.

**Table 1:** Patient demographics

Patients (*n*)	20
Age (years)	57 ± 19

Weight (kg)	88 ± 22

Height (cm)	171 ± 10

BMI (kg/m^2^)	30 ± 7

Male/female ratio	12/8

Severity of illness scores	

APACHE II	15 ± 7
SAPS II	35 ± 13
SOFA	8 ± 3

Etiology of illness (%) (*n*)	

Surgical	30% (6)
Medical	65% (13)
Trauma	5% (1)

Co-morbidities (%) (*n*)	

COPD	25% (5)
Chronic renal failure	10% (2)
Diabetes	40% (8)
Liver diseases	5% (1)
Malignancy	5% (1)
Hypertension	30% (6)
Heart disease	10% (2)
Hyperlipidemia	15% (3)

Data expressed as means ± SD or % (*n*). APACHE II, Acute Physiology and Chronic Health Evaluation, version II; SAPS II, Simplified Acute Physiology Score, version 2; SOFA, Sequential Organ Failure Assessment Score; COPD, chronic obstructive airway disease; BMI, body mass index.

GLM for repeated measures showed that for all four measures, the IAP did not change significantly over time (Huyn-Feldt test for within-subjects effects *p *> 0.34; Bonferroni-corrected pairwise comparisons, *p *≥ 0.50). Therefore, the results for all 60 sets of measurements were pooled.

**Figure 1:**
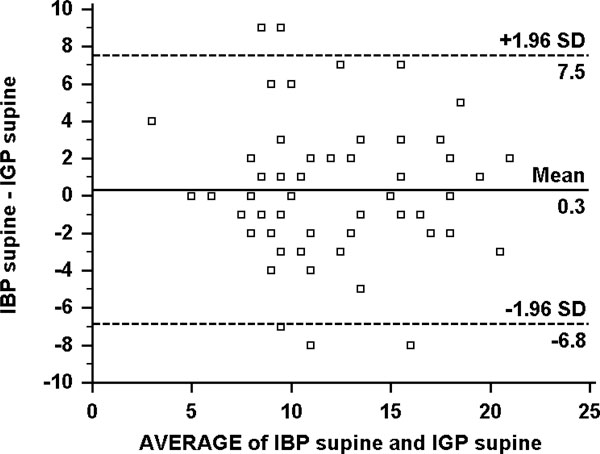
**Bland-Altman plot of the difference between IBP and IGP in the supine position**. IBP, intra-bladder pressure; IGP, intra-gastric pressure.

**Figure 2:**
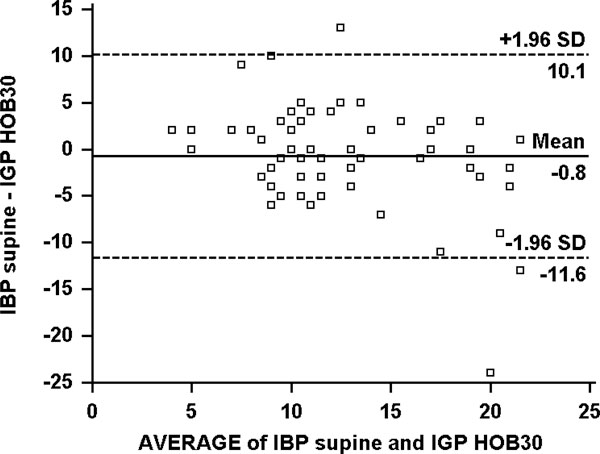
**Bland-Altman plot of the difference between IBP in the supine and IGP in the HOB30 position**. IBP, intra-bladder pressure; IGP, intra-gastric pressure.

**Figure 3:**
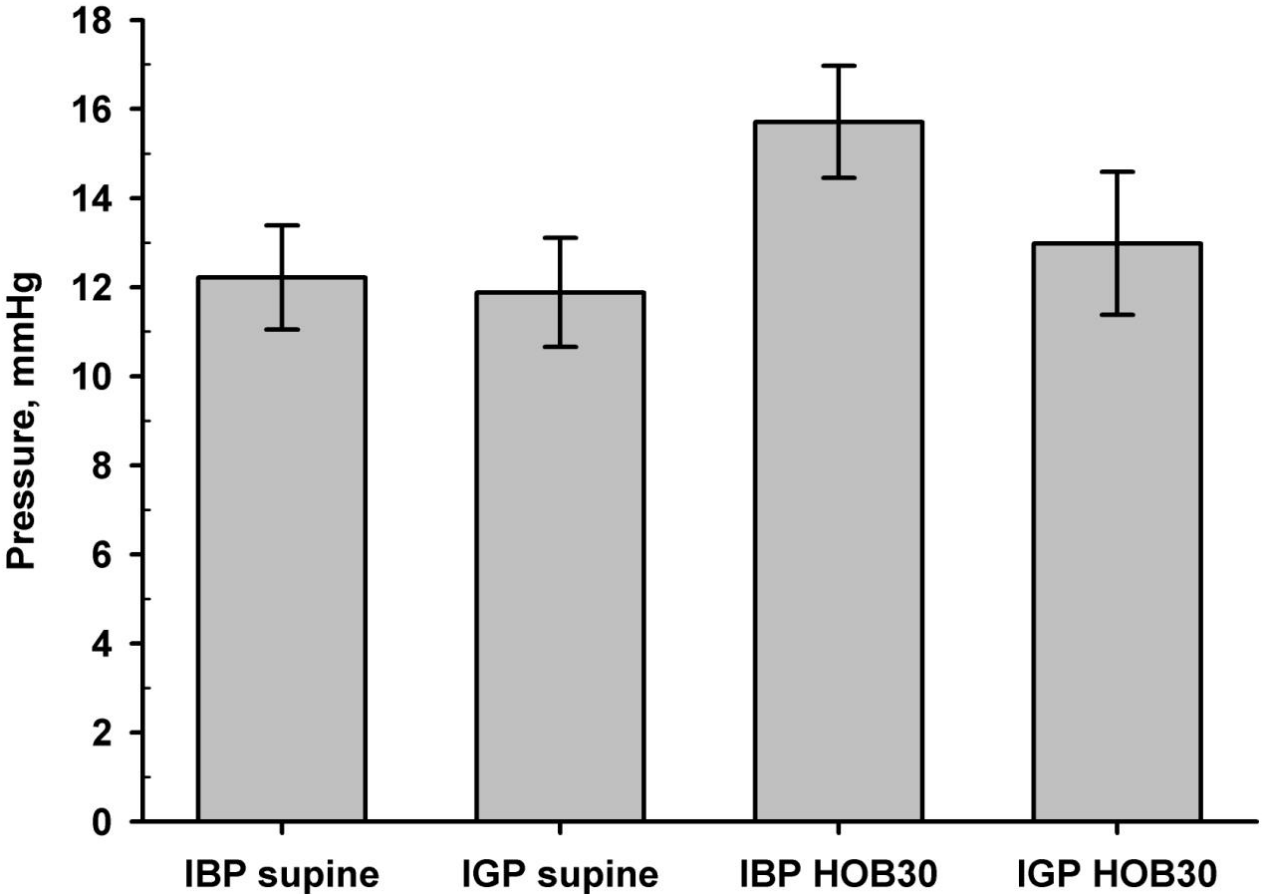
**Comparison of IBP and IGP in the supine and semi-recumbent (HOB30) position**. Mean ± SD are shown. IBP, intra-bladder pressure; IGP, intra-gastric pressure.

In the supine position, the mean IBP was 12.3 ± 4.5 mmHg compared to an IGP of 11.8 ± 4.7 mmHg (Figure 1). In HOB30 position, the mean IBP was 15.8 ± 4.9 mmHg compared to the mean IGP of 13.1 ± 6.1 mmHg (Figure 2). The mean difference in the supine position for IGP and IBP was -1.3 ± 4.6 mmHg (*p *= 0.037), whereas the mean difference in the HOB30 for IGP and IBP was -3.5 ± 3.0 mmHg (*p *< 0.001) (Figure 3). Bias, precision, limits of agreement and coefficient of determination comparing different pressure measurements are shown in Table 2.

**Table 2:** Comparison between different pressure sites and body positions

	Bias (95% CI) in mmHg	Precision (limits of agreement) in mmHg	*R*^2^	SE
IBP supine vs IGP supine	0.3 (-0.6 to 1.3)	3.7 (-6.8 to 7.5)	0.42	27.4
IBP HOB30 vs IGP HOB30	2.7 (1.3 to 4.1)	5.5 (-8.0 to 13.5)	0.28	11.2
IBP supine vs IGP HOB30	-0.8 (-2.2 to 0.7)	5.6 (-10.1 to 11.6)	0.25	39.2

Bias, precision, limits of agreement and coefficient of variation (*R*^2^) comparing different pressure measurements. IBP, intra-bladder pressure; IGP, intra-gastric pressure; HOB30, head of bed elevated at 30°; 95% CI, confidence interval; SE, standard error (2 × SD/mean IAP)

## Discussion

This study found that IGP changed to a lesser degree than IBP when changing the body position from supine to HOB30. The WSACS recommends using the IBP in the supine position to indirectly measure IAP, but gastric pressures have shown to be a valid alternative in cases where the bladder is contra-indicated. Collee et al. [15] used a simple water column technique to measure IGP in 26 intensive care patients and found a good correlation between bladder and gastric pressures. Sugrue et al. [16] showed with a modified nasogastric tube (air-filled) that in nine patients undergoing laparoscopic cholecystectomy, the IGP reflected IBP accurately (mean difference of 0.35 mmHg). Turnbull et al. [17] also used air-filled balloon catheters and compared them with direct IAP measurement. They found an estimated 2.5-mmHg difference between both techniques which is acceptable. Semi-continuous measurement of IAP using an intra-gastric compliance catheter has shown a good correlation between gastric and direct IAP measurement with a mean difference of only 0.12 mmHg and acceptable limits of agreement [18]. Similar results were published in vitro [19] and in vivo [20,21]. However, this could not be confirmed by Davis et al. [22] who demonstrated in children that the bias between nasogastric and bladder pressures was 1.3 mmHg with limits of agreement of -5.42 and 2.82. Becker et al. showed that continuous IAP measurements via the CiMON catheter (Pulsion Medical Systems, Munich, Germany) in cirrhotic patients did not correlate well with direct IAP measurements (bias of 4.9 mmHg) [23]. Finally, some animal studies have shown poor correlation between IGP and IBP [10].

Most patients in the ICU are nursed with the head of bed elevated at 30° to 45° to reduce the risk of ventilator-associated pneumonia [7,8]. Studies have shown that placing these patients in the semi-recumbent position significantly increases IAP [10]. This could lead to an over-estimation of the measured IAP.

Ideally, we need to have a method of measuring IAP in HOB30. In this study, IGP HOB30 was compared with the gold standard IAP supine via the bladder. Comparing the two methods, we found an acceptable bias, but the precision and limits of agreement exceeded the pre-defined range [14]. Therefore, based on our results, we currently cannot recommend the use of IGP in the HOB30 to be used as an alternative to IBP in the supine position.

It is uncertain if this IBP increase between supine and HOB30 is a true pressure increase throughout the entire abdomen or represents a pressure gradient within the abdomen caused by a hydrostatic fluid column or a compression of abdominal organs with a subsequent compression on the bladder or even a localized pressure difference [10].

There might have been extra-abdominal body components such as the mediastinum or lungs increasing IAP, which might have affected IGP and IBP readings to different extent. If we believe that the abdomen behaves as a hydraulic system [10], then the IBP increase between supine and HOB30 is real and clinicians should take this into account in patients with impending ACS. Further research needs to be directed towards whether the abdomen is a truly fluid container or whether it is made up of different organs with fluid and air-filled contents exerting varying pressures on these compartments as this may help explain the significance of higher semi-recumbent measurements [24].

The main limitations of our trial are that it only represented a very small group of patients. Forty percent of the patients had IAH but only one patient had ACS, and we cannot be certain that with higher IAP values the correlation between IBP in the supine position and IGP in the HOB30 might have improved. Although we did pay attention to make sure that the stomach was in a period of quiescent motor activity with no evidence of the phase 2 or 3 of the migrating motor complex, this could not be excluded entirely and may have influenced the IGP readings. Gastric motor activity was recorded by Collard and Romagnoli at a rate of about three cycles per minute [25]. Enteral feeding and ileus may also have compromised the accuracy of the IGP. However, as the IGP was lower than the IBP in the supine position, we do not think the gastric activity had a great influence on the gastric pressure. Furthermore, any residual air or residual gastric contents left in the tubing (something that is difficult to assess) might give erroneous pressure readings, hence influencing IGP. The zero reference position for measuring IGP on the mid-axillary line at the level of the xiphisternum might have influenced the readings at HOB30 when comparing with the supine position. Finally, the optimum amount of fluid to be instilled into the stomach is still unknown.

As for IBP measurements, increasing volume of fluid installed into the bladder can produce falsely elevated measurements. Therefore, the amount of 100 ml of normal saline might have been too high to adequately measure IGP. Some of these questions may be solved through bypassing the effects associated with fluid-filled systems and measuring IAP with a balloon-tipped catheter.

There are no guidelines towards critical IAP levels in patients with the HOB elevated, and it may well be that the higher readings ascertained in this position are accurate. Further research should be directed as to whether early interventions for raised IAP in the semi-recumbent position provide better outcomes in patients with ACS. Based on our results, IGP in the semi-recumbent position cannot be recommended as an estimate for IAP in supine position. Instead, IAP should be taken via an indwelling bladder catheter with the patient in a supine position to standardize values when intermittent readings are required. If readings are taken in different positions or by different routes, accuracy is reduced.

## Conclusions

Based on our results, IGP at HOB30 position cannot be used as a surrogate measure of IBP in supine position. Although previous studies have implicated that IGP and IBP can be used interchangeably, we found large limits of agreement between the two methods. IAP measured via the nasogastric tube was less influenced by position than was the IAP measured via bladder catheter. Further research is required to assess the implication of increased IAP readings in the semi-recumbent position and whether this is due to the abdomen not being a hydraulic compartment or whether this reflects a true local increase in IAP.

## Abbreviations

ACS: abdominal compartment syndrome; GLM: general linear modelling; HOB: head of bed elevation; HOB30: head of bed elevation at 30°; IAH: intra-abdominal hypertension; IAP: intra-abdominal pressure; IBP: intrabladder pressures; IGP: intragastric pressures; LA: limits of agreement; RASS: Richmond Agitation and Sedation Scale; SD: standard deviation; WSACS: World Society of Abdominal Compartment Syndrome.

## Competing interests

The authors declare that they have no competing interests.

## Authors' contributions

NR, BDK and AR participated in the design of the study. BDK and NR contributed to data collection. BDK, AR and WD performed the statistical analysis. NR and BDK drafted the manuscript. AR, NR and WD revised the manuscript. All authors read and approved the final manuscript.

## References

[B1] Malbrain MLNG Chiumello D Pelosi P Wilmer A Brienza N Malcangi V Bihari D Innes R Cohen J Singer P Japiassu A Kurtop E De Keulenaer BL Daelemans R Del Turco M Cosimini P Ranieri M Jacquet L Laterre P-F Gattinoni L Prevalence of intra-abdominal hypertension in critically ill patients: a multicentre epidemiological study Intensive Care Med 2004 30 822 829 14758472 10.1007/s00134-004-2169-9

[B2] Malbrain MLNG Chiumello D Pelosi P Bihari D Innes R Ranieri VM Del Turco M Wilmer A Brienza N Malcangi V Cohen J Japiassu A De Keulenaer BL Daelemans R Jacquet L Laterre P-F Frank G de Souza P Cesana B Gattinoni L Incidence and prognosis of intraabdominal hypertension in a mixed population of critically ill patients: a multiple-center epidemiological study Crit Care Med 2005 33 315 322 15699833 10.1097/01.ccm.0000153408.09806.1b

[B3] Cheatham ML Malbrain MLNG Kirkpatrick A Sugrue M Parr M De Waele J Balogh Z Leppaniemi A Olvera C Ivatury R D'Amours S Wendon J Hillman K Wilmer A Results from the International Conference of Experts on Intra-abdominal Hypertension and Abdominal Compartment Syndrome. II. Recommendations Intensive Care Med 2007 33 951 962 17377769 10.1007/s00134-007-0592-4

[B4] Malbrain MLNG Cheatham ML Kirkpatrick A Sugrue M Parr M De Waele J Balogh Z Leppaniemi A Olvera C Ivatury R D'Amours S Wendon J Hillman K Johansson K Kolkman K Wilmer A Results from the International Conference of Experts on Intra-abdominal Hypertension and Abdominal Compartment Syndrome. I. Definitions Intensive Care Med 2006 32 1722 1732 16967294 10.1007/s00134-006-0349-5

[B5] Cheatham ML Safcsak K Is the evolving management of intra-abdominal hypertension and abdominal compartment syndrome improving survival? Crit Care Med 2010 38 402 407 20095067 10.1097/ccm.0b013e3181b9e9b1

[B6] Malbrain MLNG Different techniques to measure intra-abdominal pressure (IAP): time for a critical re-appraisal Intensive Care Med 2004 30 357 371 14730376 10.1007/s00134-003-2107-2

[B7] Dodek P Keenan S Cook D Heyland D Jacka M Hand L Muscedere J Foster D Mehta N Hall R Brun-Buisson C Evidence-based clinical practice guideline for the prevention of ventilator-associated pneumonia Ann Intern Med 2004 141 305 313 15313747 10.7326/0003-4819-141-4-200408170-00011

[B8] Tablan OC Anderson LJ Besser R Bridges C Hajjeh R Guidelines for preventing health-care-associated pneumonia, 2003: recommendations of CDC and the Healthcare Infection Control Practices Advisory Committee MMWR Recomm Rep 2004 53 1 36 15048056

[B9] Cheatham ML De Waele JJ De Laet I De Keulenaer B Widder S Kirkpatrick AW Cresswell AB Malbrain M Bodnar Z Mejia-Mantilla JH Reis R Parr M Schulze R Puig S World Society of the Abdominal Compartment Syndrome Clinical Trials Working Group The impact of body position on intra-abdominal pressure measurement: a multicenter analysis Crit Care Med 2009 37 2187 2190 19487946 10.1097/CCM.0b013e3181a021fa

[B10] De Keulenaer BL De Waele JJ Powell B Malbrain MLNG What is normal intra-abdominal pressure and how is it affected by positioning, body mass and positive end-expiratory pressure? Intensive Care Med 2009 35 969 976 19242675 10.1007/s00134-009-1445-0

[B11] Kron IL Harman PK Nolan SP The measurement of intra-abdominal pressure as a criterion for abdominal re-exploration Ann Surg 1984 199 28 30 6691728 10.1097/00000658-198401000-00005 PMC1353253

[B12] Malbrain ML De laet I Viaene D Schoonheydt K Dits H In vitro validation of a novel method for continuous intra-abdominal pressure monitoring Intensive Care Med 2008 34 740 745 18075730 10.1007/s00134-007-0952-0

[B13] Bland JM Altman DG Statistical methods for assessing agreement between two methods of clinical measurement Lancet 1986 1 307 310 2868172

[B14] De Waele JJ Cheatham ML Malbrain MLNG Kirkpatrick AW Sugrue M Balogh Z Ivatury R De Keulenaer B Kimball EJ Recommendations for research from the International Conference of Experts on Intra-abdominal Hypertension and Abdominal Compartment Syndrome Acta Clin Belg 2009 64 203 209 19670559 10.1179/acb.2009.036

[B15] Collee GG Lomax DM Ferguson C Hanson GC Bedside measurement of intra-abdominal pressure (IAP) via an indwelling naso-gastric tube: clinical validation of the technique Intensive Care Med 1993 19 478 480 8294633 10.1007/BF01711092

[B16] Sugrue M Buist MD Lee A Sanchez DJ Hillman KM Intra-abdominal pressure measurement using a modified nasogastric tube: description and validation of a new technique Intensive Care Med 1994 20 588 590 7706574 10.1007/BF01705728

[B17] Turnbull D Webber S Hamnegard CH Mills GH Intra-abdominal pressure measurement: validation of intragastric pressure as a measure of intra-abdominal pressure Br J Anaesth 2007 98 628 634 17456490 10.1093/bja/aem060

[B18] De Waele JJ Berrevoet F Reyntjens K Pletinckx P De laet I Hoste E Semicontinuous intra-abdominal pressure measurement using an intragastric compliance catheter Intensive Care Med 2007 33 1297 1300 17522843 10.1007/s00134-007-0682-3

[B19] De Potter TJ Dits H Malbrain ML Intra- and interobserver variability during in vitro validation of two novel methods for intra-abdominal pressure monitoring Intensive Care Med 2005 31 747 751 15809871 10.1007/s00134-005-2597-1

[B20] Engum SA Kogon B Jensen E Isch J Balanoff C Grosfeld JL Gastric tonometry and direct intraabdominal pressure monitoring in abdominal compartment syndrome J Pediatr Surg 2002 37 214 218 11819201 10.1053/jpsu.2002.30257

[B21] Schachtrupp A Tons C Fackeldey V Hoer J Reinges M Schumpelick V Evaluation of two novel methods for the direct and continuous measurement of the intra-abdominal pressure in a porcine model Intensive Care Med 2003 29 1605 1608 12920511 10.1007/s00134-003-1847-3

[B22] Davis PJ Koottayi S Taylor A Butt WW Comparison of indirect methods of measuring intra-abdominal pressure in children Intensive Care Med 2005 31 471 475 15678316 10.1007/s00134-004-2539-3

[B23] Becker V Schmid RM Umgelter A Comparison of a new device for the continuous intra-gastric measurement of intra-abdominal pressure (CiMon) with direct intra-peritoneal measurements in cirrhotic patients during paracentesis Intensive Care Med 2009 35 948 952 19242674 10.1007/s00134-009-1451-2

[B24] Malbrain ML De Laet IE Willems A Van Regenmortel N Schoonheydt K Dits H Localised abdominal compartment syndrome: bladder-over-gastric pressure ratio (B/G ratio) as a clue to diagnosis Acta Clin Belg 2010 65 98 106 20491359 10.1179/acb.2010.021

[B25] Collard JM Romagnoli R Human stomach has a recordable mechanical activity at a rate of about three cycles/minute Eur J Surg 2001 167 188 194 11316403 10.1080/110241501750099357

